# Endothelial dysfunction is not a predictor of outcome in chronic obstructive pulmonary disease

**DOI:** 10.1186/s12931-020-01345-9

**Published:** 2020-04-20

**Authors:** Andreas Scherr, Desiree M. Schumann, Meropi Karakioulaki, Léo Franchetti, Werner Strobel, Michael Zellweger, Michael Tamm, Daiana Stolz

**Affiliations:** 1 Clinic of Respiratory Medicine and Pulmonary Cell Research, University Hospital of Basel and University of Basel, Petersgraben 4, 4031 Basel, Switzerland; 2 Clinic of Cardiology, University Hospital Basel, Petersgraben 4, 4031 Basel, Switzerland

**Keywords:** Arterial stiffness, COPD, Endothelial dysfunction, Flow-mediated dilation, Reactive hyperemia

## Abstract

**Background:**

Local airway inflammation may cause systemic changes which result in endothelial dysfunction. Only a few studies have used reactive hyperemia peripheral arterial tonometry (RH-PAT) in patients with chronic obstructive pulmonary disease (COPD) in order to measure their endothelial dysfunction.

**Objective:**

To determine the efficacy of endothelial dysfunction, measured by RH-PAT, in assessing disease severity and systemic burden in a cohort of COPD patients.

**Methods:**

In this prospective, monocentric study, 157 patients with moderate to very severe COPD (GOLD class II-IV) were examined for endothelial dysfunction using RH-PAT (Itamar medical Ltd., Caesarea, Israel). In a nested-cohort, examination was repeated at exacerbation. The association between reactive hyperemia index (RHI), augmentation index (AI) and disease severity and outcome parameters was analysed.

**Results:**

57% of the COPD patients had a dysfunctional endothelium and the median (IQR) RHI was 1.42 (1.27–1.53). Exacerbation of COPD was not associated with a significant change in RHI (*p* = 0.625) or ΑΙ (*p* = 0.530). None of the diagnostic or clinical outcomes of COPD was associated with RHI or arterial stiffness.

**Conclusion:**

Endothelial dysfunction is common in COPD. However, it does not seem to be a predictor neither of disease severity, nor of outcome and does not change during exacerbations of the disease.

## Background

Although preventable and treatable, chronic obstructive pulmonary disease (COPD) is one of the leading causes of mortality worldwide [1–3]. It is associated with extra-pulmonary comorbid conditions, such as peripheral vascular disease, osteoporosis, skeletal muscle dysfunction, reduced exercise capacity, erectile dysfunction, chronic depression, lung cancer and diabetes [4, 5]. These conditions are responsible for the poor outcome of advanced COPD and may reflect the systemic manifestation of COPD [6]. Cardiovascular comorbidities contribute to the morbidity and mortality of COPD and can be detected independently of traditional risk factors in about one third of COPD patients, accounting for about 50% of deaths [7]. That is because a spill-over of local airway inflammatory mediators into the systemic circulation may result in vascular changes leading to endothelial dysfunction [8]. Endothelial dysfunction may additionally be caused due to a decrease in the availability of nitric oxide (ΝΟ) and due to inflammatory-mediated changes in the structure of the vessel walls, such as the replacement of degraded elastic fibres by collagen, which increases arterial stiffness [8]. Interestingly, endothelial dysfunction is often described as a common feature of COPD and it seems to be positively correlated with the degree of airway obstruction, thus suggesting a mechanistic link between chronic airway obstruction and cardiovascular risk [9–12].

The main method of determining endothelial dysfunction in patients with COPD is by measuring the flow-mediated dilation (FMD) of the brachial artery. Reactive hyperemia-peripheral arterial tonometry (RH-PAT) is a more recent, noninvasive method to evaluate endothelial dysfunction and its use, easier and less operator-dependent, has been rapidly increasing. RH-PAT measures pulsatile volume changes in response to reactive hyperemia, a transient increase in organ blood flow that is dependent on NO synthesis and occurs after a brief period of ischemia [13–15]. RH-PAT assesses endothelial dysfunction in relation to the presence of multiple cardiovascular and metabolic risk factors [16] and can successfully predict cardiovascular events in a population with an intermediate cardiac risk profile [17]. Moreover, abnormal RH-PAT results are correlated with ischemic heart disease diagnosed using an invasive method [18].

Endothelial function assessed by digital RH-PAT is expressed as the reactive hyperemia index (RHI). The RHI describes the ratio of average amplitude during reactive hyperemia compared with the pre-occlusion baseline period [19]. A lower RHI is associated with higher complexity of coronary atherosclerotic plaque, larger systemic atherosclerotic plaque burden and adverse cardiovascular events [20–23]. Tanaka et al. [24] proposed RHI cutoff values 1.67 and 2.10 (< 1.67 for abnormal and ≥ 1.67 and < 2.10 for borderline, ≥2.10 for normal). On the other hand, the augmentation index (AI) is a measurement of arterial stiffness, which is calculated from a pulse waveform analysis of the peripheral arterial tonometry (PAT) signal at baseline and normalized to a heart rate of 75 bpm [25]. Lower AI values reflect better arterial elasticity [25].

Minet et al. investigated endothelial dysfunction in patients with COPD using RH-PAT, but their cohort consisted of only 44 patients [26]. Our objective was, therefore, to investigate endothelial dysfunction using RH-PAT in a larger and well-characterised cohort of COPD patients, so that we could determine the importance of endothelial dysfunction in assessing disease severity and systemic burden in COPD.

## Methods

Τhe PREVENT study [27] is an investigator-initiated and –driven study compiled to the Declaration of Helsinki and Good Clinical Practice Guidelines. The study was approved by the responsible ethics committee (EKNZ 306/10) and was registered at www.controlled-trials.com (identifier ISRCTN 45572998). All patients were recruited from in- and out-patients referred for clinical evaluation and lung function tests to the Clinic of Pneumology, University Hospital of Basel and provided written consent before any study assessments were initiated.

**Fig. 1:**
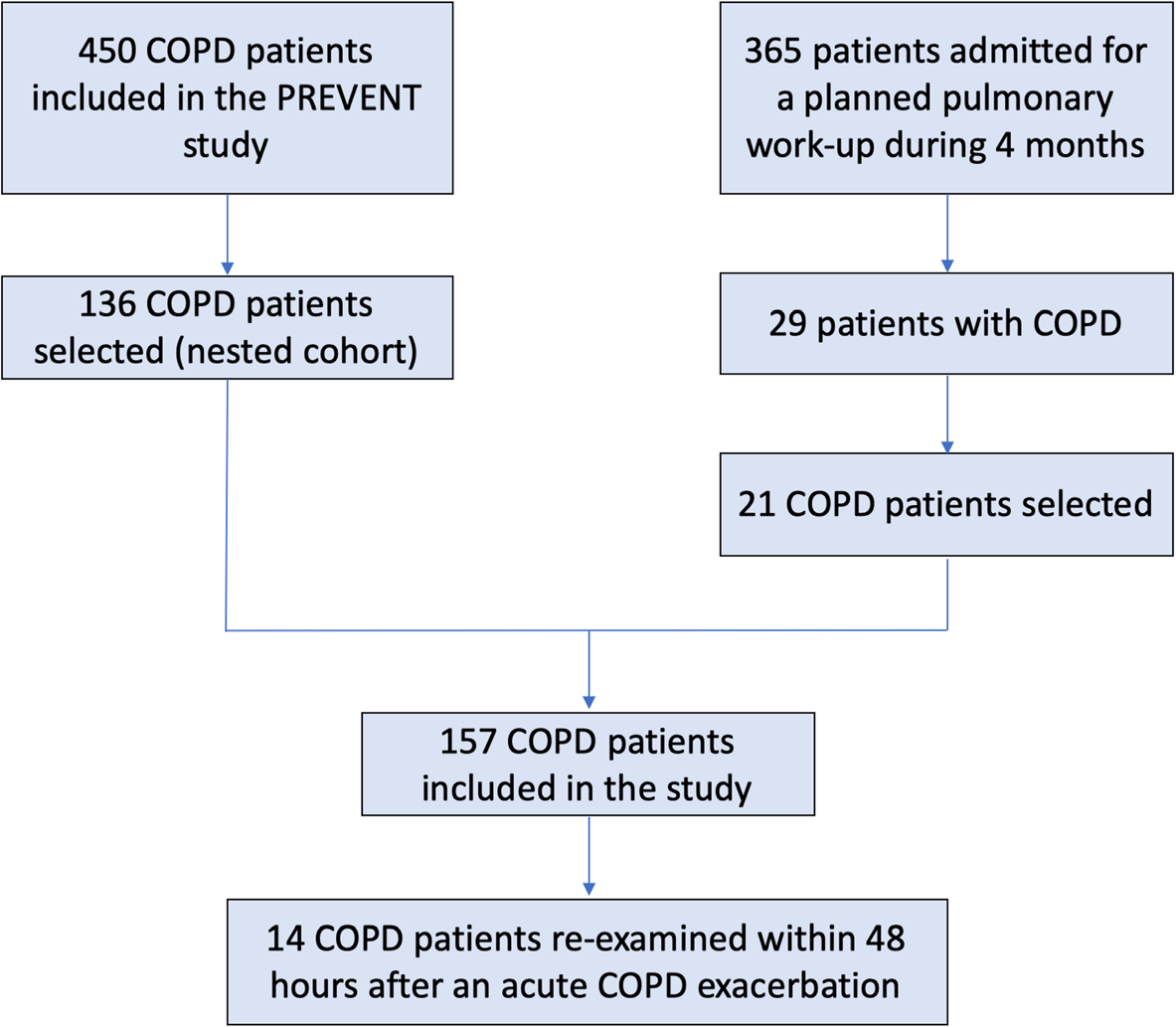
Schematic representation of the study design

The current study included a nested cohort of the PREVENT study (*n* = 136) and 21 COPD patients from a cohort of 365 patients that were admitted for a planned pulmonary workup during a period of 4 months (Fig. 1). The inclusion criteria were as follows: age ≥ 40 years; smoking history ≥10 pack-years; moderate to very severe COPD (GOLD class II-IV); clinically relevant disease, as defined by a history of exacerbations in the previous 12 months; stable disease (free of exacerbation) for ≥4 weeks prior to the start of the study. GOLD grades are based on FEV_1_% predicted: I ≥ 80%; 50% ≤ II < 80%; 30% ≤ III < 50%; and IV < 30%. The exclusion criteria were as follows: pulmonary condition other than COPD; rapid lethal disease; severe immunosuppression including manifested AIDS, organ transplantation or neutropenia (< 500 × 10^9^/L), pregnancy or breastfeeding and known allergy or intolerance to the study medicine. In addition, 14 patients were re-evaluated within 48 h after an acute exacerbation of COPD (AECOPD).

The assessments for each patient included a detailed history, blood sample collection, a physical examination and a measurement of vital signs and lung function. The 6-min walking test (6MWT) [28], the St. George’s respiratory questionnaire (SGRQ), and the COPD assessment test (CAT) were additionally required. Specifically, the 6MWT is a submaximal exercise test that entails measurement of distance walked over a span of 6 min and assesses the performance ability in a variety of cardiopulmonary diseases [29]. The SGRQ measures the impact of COPD on overall health, daily life and perceived well-being [30]. It scores from 0 to 100, with higher scores indicating more limitations [30]. The CAT is a patient-completed questionnaire, assessing the impact of COPD (cough, sputum, dyspnoea, chest tightness) on health status [31]. It scores from 0 to 40 and higher scores denote a more severe impact of COPD on a patient’s life [31].

Information regarding exacerbations were additionally acquired from the patients for the year preceding the baseline measurements and were classified as mild (requiring medical care and increased dose of short-acting β2 agonists), moderate (requiring either antibiotics and/or parenteral corticosteroids) or severe (requiring hospitalization or resulting in death).

Endothelial function was assessed by a trained and certified study nurse at scheduled and unscheduled visits, measuring reactive hyperemia with finger plethysmography, according to the recommendations of the manufacturer (Itamar medical Ltd., Caesarea, Israel) [25]. The device also measured AI.

CT-scans were analysed for emphysema and sub-classified as centrilobular emphysema and paraseptal emphysema.

For our statistical analysis, the SPSS (Statistical Package for Social Sciences) Release 22.0 software package program (SPPS, Inc., Chicago, IL) was utilized. We based our analysis on the data for FMD obtained by Moro et al. [32]. Correlation of RH-PAT values with other measurable disease-relevant parameters was analysed using the Spearman rho coefficient. A comparison of RH-PAT values during stable disease and AECOPD in the same 14 individuals (paired samples) was performed using Mann-Whitney analysis. The predictive value of RH-PAT for adverse outcome events (AECOPD, hospitalisations, cardiovascular events, death) was analysed by uni- and multivariable cox-regression proportional analysis. Adjustment for confounding variables like age, gender and cardiovascular risk factors was pre-defined according to the literature [26]. All tests were two-tailed and the level of statistical significance was set at 0.05. Results were expressed as mean (standard deviation) or median (interquartile range), unless otherwise stated.

## Results

In this study, we included 157 patients with stable COPD (Fig. 1), 69% of which were male and 42% were smokers (Table 1). The patients were classified as follows: 68% GOLD II, 22% GOLD III, and 10% GOLD IV. The most common cardiovascular comorbidities were arterial hypertension (64%) and coronary artery disease (28%). Accordingly, 42% were on statin medication and 56% were taking aspirin.

**Table 1:** Baseline characteristics of the patients included in the study

Parameter	COPD patients (***n*** = 157) n (%); median (IQR)
Male	108 (69)
Age (years)	67 (59–73)
Current smoker	65 (42)*
Pack years	53.1 (25)
BMI (kg/m^2^)	26 (23–31)
BODE Index	2 (3)
6MWT (m)	420 (154)
Exacerbation rate	1 (0)
Pulse (bpm)	74 (67–83)
Systolic blood pressure (mmHg)	133 (121–146)
Diastolic blood pressure (mmHg)	74 (63–82)
Unadjusted Charlson Score	2 (2)^c^
MMRC score	2 (1)^a^
COPD Assessment Score	16 (10)^b^
**Lung function**	
FEV_1_ (L)	1.3 (0.9–1.7)^d^
FVC (L)	2.8 (2.3–3.4) ^d^
FEV_1_, % predicted	52.7 (40.1–65.6) ^d^
FVC, % predicted	82.3 (71.8–99.6) ^d^
TLC (L)	6.7 (5.6–7.8)^e^
TLC, % predicted	111.0 (98.3–129.6)^e^
DLCO_SB (mmol/min/kPa)	4.5 (3.6–5.8)^a^
DLCO_SB, % predicted	56.7 (46.5–69.8)^a^
DLCO/VA (mmol/min/kPa)	0.91 (0.74–1.16)^a^
DLCO/VA, % predicted	66.3 (54.3–87.7)^a^
**SGRQ**	
Symptom score	42.5 (24.7–67.0)
Activity score	53.8 (41.4–71.0)
Impact score	19.5 (12.7–36.6)
Total score	34.2 (23.7–51.5)
**Imaging:** CT-scans	126 (80)
Emphysema	94 (75)
Centrilobular emphysema	79 (84)
Paraseptal emphysema	20 (21)

*BMI* Body mass index, *BODE* BMI, airflow obstruction, dyspnea and exercise capacity, *6MWD* 6-min walk distance, *MMRC* Modified Medical Research Council, *FEV*_*1*_ Forced expiratory volume in 1 s., *FVC* Forced vital capacity, *TLC* Total lung capacity, *DLCO* Diffusing capacity of the lung for carbon monoxide, *SGRQ* St. George’s Respiratory Questionnaire *2 missing values; ^a^ 7 missing values; ^b^ 21 missing values; ^c^ 22 missing values; ^d^ 1 missing value; ^e^ 7 missing values

Additionally, 57% of the COPD patients had a dysfunctional endothelium. The median (IQR) RHI score was 1.42 (1.27–1.53) and none of the diagnostic or clinical outcomes of COPD was associated with the RHI or arterial stiffness (Table 2). The RHI had no significant effect on time to exacerbation up to 3.3 years after inclusion in the study (Hazard Ratio = 1.001, 95% CI 0.436–2.300, *p* = 0.998).

**Table 2:** Linear regression looking at the effect of various variables on RHI values

	Beta	95% Confidence Interval		***P***-value
		Lower limit	Upper limit	
Age	−0.147	−0.021	0.001	0.066
BMI	−0.259	−0.041	− 0.011	**0.001**
Systolic blood pressure	0.056	−0.004	0.008	0.514
Diastolic blood pressure	−0.008	−0.010	0.009	0.923
Heart rate	−0.077	−0.011	0.004	0.371
Gender	0.262	0.146	0.556	**0.001**
Smoking status	0.031	−0.163	0.241	0.706
FEV_1_ (L)	0.003	−0.154	0.159	0.977
BODE index	−0.011	−0.051	0.044	0.887
BORG	−0.104	−0.096	0.026	0.262
CAT Score	−0.055	−0.020	0.010	0.524
6MWT	0.027	−0.001	0.001	0.771

*BMI* Body mass index, *FEV*_*1*_ Forced expiratory volume in 1 s., *BODE* BMI, airflow obstruction, dyspnoea and exercise capacity, *BORG* Borg dyspnoea scale, *CAT score* COPD assessment score, *6MWD* 6-min walk distance

Significantly, more male than female and more patients with diabetes as a comorbidity had a dysfunctional endothelium (Table 3). The COPD patients with dysfunctional endothelium had significantly higher BMI than those with normal endothelial function. There were no other differences between the two groups of patients and no association with other cardiovascular comorbidities. Moreover, there was no association between RHI and aspirin (Beta = − 0.052, 95% CI -0.271 – 0.140; *p* = 0.530) nor between endothelial dysfunction and aspirin (OR = 1.342, *p* = 0.378).

**Table 3:** COPD patients with dysfunctional endothelium compared to COPD patients with normal endothelial function

Parameter	Dysfunctional endothelium, ***n*** = 90 n (%); median (IQR)	Normal endothelial function, ***n*** = 67 n (%); median (IQR)	***P***-value
Male	70 (65)	38 (35)	**0.005**
Age (years)	67 (61–75)	66 (57–72)	0.169
Current smoker	38 (58)	27 (42)	0.515
Pack years	50 (40–63)	50 (40–70)	0.627
BMI (kg/m^2^)	28 (25–33)	24 (21–27)	**< 0.001**
6MWT (m)	410 (315–490)	425 (340–480)	0.414
Exacerbation rate in the previous year	1 (0)	1 (0)	0.473
Heart rate (bpm)	75 (67–83)	73 (67–84)	0.770
Systolic blood pressure (mmHg)	132 (119–145)	133 (124–147)	0.422
Diastolic blood pressure (mmHg)	74 (63–82)	72 (63–80)	0.295
FeNO (ppm)	16 (11–26)	19 (13–25)	0.261
Unadjusted Charlson Score	2 (1–3)	2 (1–2)	0.192
CAT Score	17 (11–21)	15 (11–21)	0.717
**Lung Function (post-brd)**			
FEV_1_, in L	1.5 (1.2–2.1)	1.4 (0.94–1.7)	**0.032**
FVC, in L	3.0 (2.5–3.7)	2.9 (2.4–3.4)	0.159
FEV_1_, % predicted	59.0 (44.2–69.6)	55.9 (39.0–69.0)	0.469
FVC. % predicted	90.1 (73.8–102.4)	91.0 (76.0–105.2)	0.493
FEV1/FVC %predicted	47.5 (40.8–60.3)	47.1 (34.4–56.1)	0.237
Reactive hyperemia index	1.42 (1.27–1.53)	2.12 (1.97–2.48)	**0.000**
**BODE Index**			0.872
≤ 2	48 (54)	35 (51)	
≥ 3	41 (46)	33 (49)	
GOLD Grade*			0.862
GOLD II	61 (68.5)	45 (66.2)	
GOLD III	21 (23.6)	15 (22.0)	
GOLD IV	7 (7.9)	8 (11.8)	
**mMRC score**			0.613
≤ 2	51 (56)	40 (44)	
≥ 3	36 (61)	23 (39)	
**SGRQ**			
Symptoms score	47 (25–67)	41 (23–69)	0.771
Activity score	54 (41–72)	57 (37–66)	0.961
Impact score	19 (13–39)	20 (11–36)	0.610
Total score	34 (25–53)	33 (23–49)	0.689
**Comorbidities**			
Diabetes	19 (86)	3 (14)	**0.003**
Pulmonary arterial hypertension	6 (43)	8 (57)	0.274
Renal disease	11 (50)	11 (50)	0.495
Arterial hypertension	61 (60)	40 (40)	0.208
Congestive heart failure	12 (52)	11 (48)	0.609
Coronary artery disease	29 (66)	15 (34)	0.146
Cerebrovascular disease	8 (57)	6 (43)	0.971
Myocardial infarction	11 (55)	9 (45)	0.870
**Medication**			
SHB2AC	7 (50)	7 (50)	0.385
SABA	31 (65)	17 (35)	0.470
LABA/ICS	81 (60)	55 (40)	0.648
LABA	12 (75)	4 (25)	0.209
LAMA	81 (60)	54 (40)	1.000
Mucolytics/Antioxidants	10 (59)	7 (41)	0.863
Statins	39 (63)	23 (37)	0.223
Aspirin	50 (60)	33 (40)	0.377
Ace inhibitor	46 (64)	26 (36)	0.127

*BMI* Body mass index, *6MWD* 6-min walk distance, *CAT score* COPD assessment score, *brd* bronchodilator, *FEV*_*1*_ Forced expiratory volume in 1 s., *FVC* Forced vital capacity, *BODE* BMI, airflow obstruction, dyspnoea and exercise capacity, *GOLD* Gold Initiative for Chronic Obstructive Lung Disease, GOLD grades are based on FEV1% predicted: 50% ≤ II ≤ 80%; 30% ≤ III ≤ 50%; and IV ≤ 30%, *mMRC* Modified Medical Research Council Dyspnoea scale, *SGRQ* St. George’s Respiratory Questionnaire, *SHB2AC* Short-acting beta 2 agonist plus anticholinergic, *SABA* Short-acting beta 2 agonist, *LABA/ICS* Long-acting beta 2 agonist plus glucocorticosteroids, *LABA* Long acting beta 2 agonist, *LAMA* Long-acting muscarinic antagonist

There was no significant difference in RHI between patients with emphysema and those without (median 1.6 vs. 1.6, *p* = 0.89), with centrilobular emphysema or without (median 1.6 vs. 1.7; *p* = 0.87) nor with paraseptal emphysema or without (1.6 vs. 1.7; p = 0.89). Linear regression adjusting for confounding factors such as age, BMI, gender, statin use, diabetes, arterial hypertension, FEV_1_, smoking status, and pack years, showed no association between emphysema, any type of emphysema and RHI (Table 4). A chi-squared test showed no association between emphysema (*p* = 0.85), centrilobular emphysema (*p* = 0.94), paraseptal emphysema (*p* = 0.32) and the absence or presence of endothelial dysfunction.

**Table 4:** Association of emphysema with reactive hyperemia index and arterial stiffness

	Univariate analysis		Multivariate analysis^**a**^	
	Beta-estimate ± SEM	***P***-value	Beta-estimate ± SEM	***P***-value
**RHI**				
Emphysema	0.034 ± 0.13	0.78	−0.05 ± 0.15	0.74
Centrilobular emphysema	0.08 ± 0.19	0.70	0.10 ± 0.19	0.60
Paraseptal emphysema	0.03 ± 0.06	0.68	0.04 ± 0.06	0.48
**Arterial stiffness**				
Emphysema	1.8 ± 3.8	0.64	3.2 ± 4.3	0.457
Centrilobular emphysema	0.3 ± 5.0	0.95	−6.4 ± 5.1	0.210
Paraseptal emphysema	**4.7 ± 1.6**	**0.005**	**4.3 ± 1.5**	**0.006**

^a^Model adjusted for age, gender, body mass index, smoking status, pack years, FEV_1_, arterial hypertension, diabetes mellitus, and statin use

**Fig. 2:**
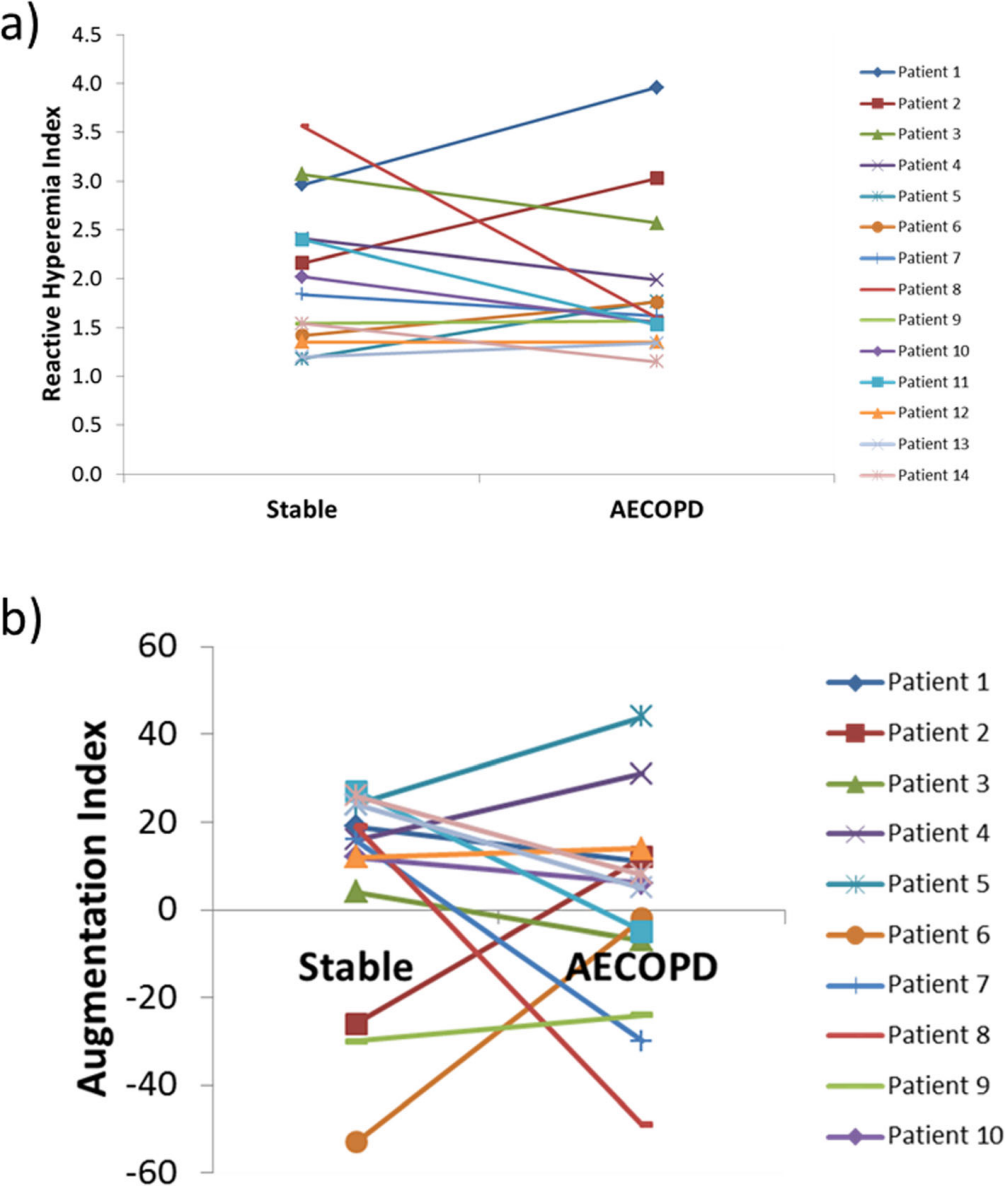
**a** Reactive hyperemia index (RHI) and (**b**) arterial stiffness, as measured by the augmentation index (AI) remained unchanged during stable state and exacerbation, as assessed by the paired t-test (*p* = 0.625 and *p* = 0.530 accordingly)

Arterial stiffness, as represented by AI, was 10.50 in COPD patients. The factors associated with arterial stiffness were age (Spearman rho = 0.236; *p* = 0.001), heart rate (Spearman rho = − 0.406; *p* < 0.001) and systolic blood pressure (Spearman rho = 0.273; p < 0.001). The predictors for arterial stiffness in patients with COPD were heart rate (Beta = − 0.409, 95% CI -0.862 - -0.408, p < 0.001), systolic blood pressure (Beta = 0.346, 95% CI 0.187–0.506; p < 0.001), BMI (Beta = − 0.175, 95% CI -0.944 - -0.89; *p* = 0.018) and gender (Beta = 0.264, 95% CI 4.911–16.679; p < 0.001). Arterial stiffness had no effect on time to exacerbation (Hazard ratio = 0.996, 95% CI 0.975–1.017, *p* = 0.706). Interestingly, there was no significant difference in RHI (*p* = 0.625, Fig. 2a) and AI (*p* = 0.530, Fig. 2b) when we compared patients during a stable phase of COPD and the same patients during an acute exacerbation of COPD.

Arterial stiffness did not change significantly between patients with emphysema compared to those without emphysema (median 11 vs. 10: *p* = 0.46), with centrilobular emphysema and those without (10 vs. 13; *p* = 0.65) and between those with paraseptal emphysema and those without (9 vs. 11; *p* = 0.88). A significant association was seen in univariate and multivariate analysis between arterial stiffness and paraseptal emphysema (Table 4).

## Discussion

Up-to-date, this is the largest study evaluating endothelial dysfunction in COPD and the first to evaluate potential changes in endothelial dysfunction between stable state and exacerbation of COPD using RH-PAT. We found that 57% of the patients with COPD had endothelial dysfunction. This is comparable to the results of Minet et al. [26], who indicated endothelial dysfunction in 50% of their COPD patients. Malerba et al. [33] reported a higher incidence of endothelial dysfunction (75%) in COPD patients and a lower RHI score (1.31). This might potentially be attributable to the small sample size of their study (*n* = 16).

Similar to the vast majority of COPD patients, our patients had several cardiovascular comorbidities. Indeed, we found an association between endothelial dysfunction and diabetes mellitus. However, we found no association between dysfunctional endothelium and other cardiovascular comorbidities or aspirin, a medication considered to influence endothelial function [34]. This is surprising and could point to a technical fault, yet, the RHI measured for patients with arterial hypertension (RHI = 1.8 ± 0.7) was similar to that reported by Weisrock et al. (RHI = 1.7 ± 0.4 to 1.8 ± 0.4) [35]. Additionally, we confirmed the finding of van der Heijden et al. [36] that BMI is associated with endothelial dysfunction. On the other hand, more than half of the patients assessed in our study had arterial hypertension. Weisrock et al. [35] indicated that PAT is not a reliable method for measuring endothelial dysfunction in patients with arterial hypertension. This could, therefore, explain why we found no association between RHI and most cardiovascular comorbidities or aspirin in our study.

Unlike Marchetti et al. [37], Ozben et al. [38] and Urban et al. [39], in our study endothelial dysfunction was similar at stable state and exacerbation of COPD. This finding is rather intriguing, as exacerbations of COPD are linked to bacterial and viral infections, increase in local and systemic inflammation, hypoxia, pulmonary arterial hypertension and congestive heart failure; all these conditions are independently related to endothelial dysfunction [40–42]. Furthermore, there is a well-known increase in cardiovascular risk associated with exacerbation of COPD [43, 44]. On the other hand, although Marchetti et al. [37] and Ozben et al. [38] found lower FMD during exacerbation compared to the recovery period, they found no difference between patients with COPD in recovery and controls. They also found no difference in brachial artery diameter during exacerbation and recovery and no association between FMD and lung function [10, 37, 38]. That said, considering the small sample size used for the exacerbation analysis, it would be prudent to look at endothelial dysfunction before and after exacerbation in a larger cohort.

In two previous studies, Minet et al. [26] and Malerba et al. [33] found an association between RHI and FEV_1_. However, the current results suggest no association between RHI and any COPD parameter i.e. airflow limitation, exercise capacity, BODE index, GOLD status or modified Medical Research Council (mMRC) dyspnoea scale. These differences could lie in the fact that both previous studies had relatively small populations (*n* = 44 and *n* = 16, respectively). In addition, the discrepancies in the findings may be attributed to the fact that: 1) our study only excluded COPD patients with other pulmonary conditions, rapid lethal disease and severe immunosuppression and therefore affords a more reasonable representation of the general COPD population, while, on the other hand, Malerba et al. [33] excluded patients with a history of any cardiovascular disease except arterial hypertension, diabetes mellitus and if the patient had an exacerbation in the previous 6months; and 2) in the study of Malerba et al. [33], patients had less severe COPD than the patients in our study. In a number of studies, both FMD and RHI are lower in COPD patients than in controls, but the association with COPD parameters is tenuous [45, 46]. Eickhoff et al. [10] found an association between FMD and FEV_1_% predicted in a univariate analysis, but no significant association between FMD and GOLD status. Moro et al. [32] found an association between FMD and airflow limitation (FEV_1_/VC), however, they did not measure or adjust for brachial artery diameter. Barr et al [47] found an association between FMD and FEV_1_, but this association was present both in COPD and non-COPD patients. Clarenbach et al. [48] demonstrated that a change in FMD was associated with a change in FEV_1_ in COPD patients after lung volume reduction surgery. However, they did not look at the relationship between FMD and FEV_1_ at baseline, nor did they take COPD medication into account. Importantly, Barr et al. [47] found that COPD medication is associated with large differences in FMD.

Unlike Bhatt et al. [49], we found no association between RHI or AI and centrilobular emphysema. This could be because coronary calcification, which was used in the analysis by Bhatt et al. [49], is an independent process with different pathways compared to endothelial dysfunction [50]. There was, however, an association between paraseptal emphysema and arterial stiffness. Patients with paraseptal emphysema are at higher risk of developing lung cancer [51]. Arterial stiffness is associated with haematological malignancies [52], but its role in lung cancer is unknown.

Our study has several limitations. It was a monocentric study including patients with COPD receiving extensive treatment for their disease. In addition, we did not exclude patients with cardiovascular comorbidities. Strengths included the full characterisation of the study population, the multivariable adjustment for several confounding factors and the inclusion of a large COPD population with commonly encountered comorbidities, which assures the generalizability of our results.

## Conclusions

In conclusion, we found that 57% of patients with COPD had an incidence of endothelial dysfunction with a median (IQR) RHI of 1.42 (1.27–1.53). However, RHI was not associated to any COPD-related outcome and endothelial dysfunction was similar at stable state and at exacerbation of COPD. In addition, endothelial dysfunction, as measured using RH-PAT, is neither a predictor of disease severity nor outcome in COPD.

## Data Availability

The datasets used and analysed during the current study are available from the corresponding author on reasonable request.

## Abbreviations

6MWT: 6-min walking test
6MWD: 6-min walking distance
AI: Augmentation index
BMI: Body mass index
COPD: Chronic obstructive pulmonary disease
FEV_1_: Forced expiratory volume in 1 s
FMD: Flow-mediated dilation
RHI: Reactive hyperemia index
RH-PAT: Reactive hyperemia peripheral arterial tonometry
